# Is Quarter of Birth a Risk Factor for Developmental Coordinator Disorder in Preschool Children?

**DOI:** 10.3390/ijerph18115514

**Published:** 2021-05-21

**Authors:** Rubén Navarro-Patón, Silvia Pueyo Villa, Juan Luis Martín-Ayala, Mariacarla Martí González, Marcos Mecías-Calvo

**Affiliations:** 1 Facultad de Formación del Profesorado, Universidade de Santiago de Compostela, 27001 Lugo, Spain; ruben.navarro.paton@usc.es; 2 Facultad de Ciencias Sociales y Humanidades, Universidad Europea del Atlántico, 39011 Santander, Spain; silvia.pueyo@uneatlantico.es; 3 Coordinación de posgrado, Universidad Internacional Iberoamericana, 24560 Campeche, Mexico; juan.martin@uneatlantico.es; 4 Facultad de Ciencias de la Salud, Universidad Europea del Atlántico, 39011 Santander, Spain; mariacarla.marti@uneatlantico.es; 5 Centro de Investigación y Tecnología Industrial de Cantabria (CITICAN), 39011 Santander, Spain

**Keywords:** relative age effect, childhood, MABC-2, motor competence

## Abstract

The purpose of this study was to determine the probability that preschool children have severe motor difficulties or are at risk of motor difficulties, according to quarter of birth and gender. Five hundred and eighty-eight preschool-age children were evaluated, of which 318 (54.08%) were boys and 270 (45.92%) were girls, with a mean age of 4.66 years (SD = 0.53). The Movement Assessment Battery for Children-2 (MABC-2) was used to collect the data. The results obtained were the following: Regarding students with severe motor difficulties: 6.7% born in quarter 1 (Q1); 13.3% born in the second quarter (Q2); 20.0% born in the third quarter (Q3); and 60.0% born in the fourth quarter (Q4). The probabilities found (OR) were: Q1 vs. Q2 (OR = 3.15; *p* < 0.05); Q1 vs. Q3 (OR = 4.68; *p* < 0.005); Q1 vs. Q4 (OR = 12.40; *p* < 0.001); Q2 vs. Q4 (OR = 4.04; *p* < 0.001); and Q3 vs. Q4 (OR = 2.65; *p* < 0.005). The adjusted ORs, with respect to the probabilities of having severe motor difficulties, were the following: Being born in Q4 is 13.03 times more likely than being born in Q1 (*p* < 0.001); those born in Q3 are 4.85 times more likely than those born in Q1 (*p* < 0.05); and those born in Q2 4.14 times more than those born in Q1 (*p* < 0.05). The conclusion is that children born in Q4 are more likely to be classified as children with severe difficulties compared to children born in the other quarters of the same year.

## 1. Introduction

The acquisition of adequate motor competence (MC) during childhood is crucial for the physical, social, and cognitive development of children [1,2,3,4], of which a delay can have lasting negative effects [5] such as developmental coordination disorder (DCD), defined as a poor ability to perform or learn age-appropriate motor skills [6]. However, despite the importance of MC in the development of the daily life of preschool children, there are studies that show that its lack of development creates motor delays in this stage [7]. Thus, MC should be one of the most important contents to be developed in this educational stage [8,9] to provide quality Physical Education (PE) [10,11].

In Spain, as in many other countries, school is accessed by chronological age groups [12]. In this way, it is about ensuring an educational process that is as uniform and appropriate as possible, providing equal opportunities to all boys and girls [13]. This type of grouping can cause the opposite effect when being evaluated in subjects such as (PE), which do not take into account the chronological age difference [14], nor possible differences in maturity and experience among the children of the same cohort [15]. This chronological age difference between subjects of the same age group is known as relative age [15], and its consequence is the Relative Age Effect (RAE) [13,16].

Studies on RAE and MC in preschool children have been scarce so far, but existing ones indicate that this effect gives an advantage to those born in the first quarters of the year, presenting better MC than those born in the last quarters of the year, by the mere fact of having been born before [17,18,19]. For this reason, this effect should be taken into account in the assessment of MC within PE classes, since, as a general rule, standardized tests are usually used [1,20]. These tests can cause biasness by reinforcing the competence of more mature and older children [21,22]. When gender is added to the MC, the tendency is for boys to have better MC compared to girls [23,24,25,26]. In this sense, boys tend to show higher performance in gross motor skills (control and manipulation of objects) and general MC compared to girls [23,24,25,26,27,28,29,30,31], while girls show better performance in fine motor skills [23,25,31,32,33,34,35] and balance [24,25,31,32,33,35,36], although there are also studies in which no differences were found based on gender [37,38].

For the MC evaluation, one of the most used batteries is the Movement Assessment Battery for Children-2 (MABC-2) [39], which has been recommended by the European Academy of Childhood Disability to detect the delay of MC in children [40]. Through this standardized battery, lower percentiles than those expected for their chronological age can be detected in preschool children. According to the MABC-2 battery manual, one of its objectives is to identify motor development problems [39]. The MABC-2 battery consists of a model that evaluates MC in three different motor domains: Manual dexterity (MD), aiming and catching (A&C), and balance (Bal) through which scalar scores are obtained that enable calculating a total scalar score and, through it, the total battery percentile. Based on the total percentile of the MABC-2 test, a “traffic light system” identifies a child’s MC as belonging to one of three categories: No motor difficulties (green), at risk of motor difficulties (amber), and severe motor difficulties (red). This last category (red = severe motor difficulties) is associated with the confirmation of a developmental coordination disorder (DCD) [6].

Research has been developed on motor competence in early ages, RAE, and gender; however, there are few studies that investigate the influence of relative age on MC, performance measures [17,18,41,42], and probability of obtaining percentiles lower than those expected by age in MC.

For all the above, the main purpose of this study was to know the probability that preschool children present severe motor difficulties or are at risk of suffering motor difficulties, according to the trimester of birth and gender. The secondary objectives were (1) to know if there are differences between the quarter of birth and the CM of preschool children and (2) to establish if there are differences in the global competence (total percentile) among boys and girls.

## 2. Materials and Methods

### 2.1. Study Design

A cross-sectional descriptive observational study was carried out with data collection through the MABC-2 battery.

The main dependent variable of the study was the classification of preschool children following the traffic light system provided by the MABC-2 battery (that is, red: Children with severe motor difficulties; amber: Children at risk of suffering motor difficulties; and green: Children without motor difficulties).

Gender (male vs. female) and quarter of birth (Q1. (January–March); Q2. (April–June); Q3. (July–September); Q4. (October–December)) were used as independent variables.

### 2.2. Study Population

The study was carried out in Galicia (Spain) with a non-probabilistic sample belonging to nine educational centers, selected for geographical proximity and ease of access to the sample.

A total of 628 preschool children between 4 and 5 years old were invited, of which 40 were excluded for not providing the informed consent signed by their parents or legal guardians. In the end, the sample consisted of 588 preschool children.

All preschool children were classified according to their quarter of birth (quarter 1 (Q1: born from January to March); quarter 2 (Q2: born from April to June); quarter 3 (Q3: born from July to September) and quarter 4 (Q4: born from October to December)), and by gender group (boys and girls).

### 2.3. Tool

For the data collection, the MABC-2 battery adapted to the Spanish context was used [43]. It has proven to be a valid and reliable test to measure changes in MC over time [39,43,44] in children of different ages, with very high inter-rater reliability [45]. This battery consists of a three-factor model in which MC is assessed: Manual dexterity (MD, 3 tests), aiming and catching (A&C, 2 tests), and balance (Bal, 3 tests). Through the scalar scores of the 8 tests, the total scalar score (range 1–19) is obtained, and based on this score, the total percentile score (0.1–99.9) is obtained. Based on the total percentile, preschool children are classified using a “traffic light system” that identifies the child’s MC as belonging to one of three categories: Without motor difficulties (green: ≥16th percentile), at risk of suffering motor difficulties (amber: Between 6th and 15th percentiles, both included), and with severe motor difficulties (red: ≤5th percentile).

### 2.4. Procedure

First, the educational centers were contacted to request their collaboration. For this, the procedures and objectives to be carried out were explained to them. Once the approval of the centers was obtained, the teachers were also informed. Subsequently, an information sheet about the study and informed consent were sent to the parents or legal guardians of the minors. Once the sheet was read and signed by the parents or legal guardians, the measurements began.

On the one hand, the necessary sociodemographic data (age, date of birth, gender) were collected and, on the other, the MC evaluation was started by using the MABC-2 battery. For this, each child was individually evaluated by an expert examiner, who always assessed the same test following the same methodology in all measurements according to the MABC-2 battery examiner’s instruction manual.

Before beginning the tests, a practical test was allowed to be carried out on the preschool children, with the possibility of correction by the examiner. Once the tests began, no instructions were given.

At the end of the tests, the following data were obtained: (1) The scalar scores for each of the tests; (2) the scalar and percentile scores for each of the three dimensions (that is, MD, A&C, and Bal); and (3) total and scalar scores with their equivalent percentiles.

Once the total percentile was obtained, the preschool children were classified according to their equivalence with the MABC-2 battery traffic light system (i.e., green, amber, or red).

### 2.5. Ethics

The research protocol was approved according to the Declaration of Helsinki, by the Ethics Committee of the national platform Educa with the code number 22019.

### 2.6. Statistical Analysis

The preschool children’s characteristics are presented in frequency tables as a percentage of categorical data (i.e., gender) and with median values with interquartile range for continuous data (i.e., age). To compare the differences between groups, two-tailed tests were used. Categorical variables were analyzed with the chi square test. Logistic regression was used to evaluate trends in the quarter of birth with the cataloging of students and gender. Therefore, the association between birth month, gender, and traffic light outcome (green, amber, and red) was examined. Associations are presented as odds ratios (OR) with 95% confidence intervals (CI). All data were processed using the SPSS version 25 statistical package (SPSS Inc., IBM, Chicago, IL, USA) for MS Windows. 

## 3. Results

Table 1 presents the basic characteristics of the sample. Preschool children with severe motor difficulties (red) have a lower age average, are mostly boys, and are born in the last quarters of the year, with respect to the green and amber groups.

### 3.1. Global Assessments

Depending on the quarter of birth, preschoolers born in Q4 are more likely to be identified with severe motor difficulties (red = DCD) (OR = 12.40), or at risk of suffering motor difficulties (amber) (OR = 4.795), compared to their peers born in Q1 (Table 2).

On the other hand, preschool children born in Q3 are also more likely to be identified as at risk for motor difficulties (amber) compared to their peers born in Q1 (OR = 4.53) and Q2 (OR = 3.42).

### 3.2. Gender

Boys born in Q4 are more likely to be identified with severe motor difficulties (red = DCD) (Q4 vs. Q1, OR = 1.41; Q4 vs. Q2, OR = 1.88; Q4 vs. Q3, OR = 1.99) or at risk of motor difficulties (amber) (Q4 vs. Q1, OR = 5.81; Q4 vs. Q2, OR = 5.30; Q4 vs. Q3, OR = 4.28) than those born in the other quartiles (Table 3).

Girls born in Q4 are more likely to be identified with severe motor difficulties (red = DCD) than girls born in Q1 (Q4 vs. Q1, OR = 2.45). However, girls born in Q3 are more likely to be identified as at risk for motor difficulties (amber) than those born in Q4, compared to those born in Q1 (Q3 vs. Q1, OR = 3.63; Q4 vs. Q1, OR = 3.55) (Table 3).

### 3.3. Quarter of Birth and GENDER

In general, boys have a statistically significant association of being identified with severe motor difficulties (red = DCD), compared to girls, but not for boys born in Q1, which is significantly associated with being identified without having motor difficulties (green) (Table 4).

### 3.4. Explanatory Logistic Regression Model

After applying a logistic regression model (Table 5), preschool children born in Q4 and Q3 are significantly associated with being identified with red (Q4: OR = 13.028; Q3: OR = 4.852) or amber (Q4: OR = 4.832; Q3: OR = 4.624) compared with the rest of the quarters and gender.

On the other hand, being a boy is significantly associated with the color green (OR = 2986).

## 4. Discussion

The purpose of this study was to determine the probability that preschool children present motor difficulties, according to the quarter of birth and gender. Thus, according to the overall results obtained, preschool children born in Q4 are up to 12 times more likely than their peers born in Q1 to be identified with severe motor difficulties when taking the MABC-2 tests. This fact is maintained after the application of a regression model in which being born in Q4 is positively related to being identified with severe motor difficulties (red = DCD).

Within the same class, there may be children with up to almost 12 months of chronological age difference [14], depending on the quarter of birth, and there may be differences in maturation and practical experience between them [15]. This age difference is precisely what is evidenced in our study, and what could make preschool children born in Q4 present higher rates of scores below the fifth percentile, and therefore have a higher probability of being identified as children with severe motor difficulties (red = DCD), as occurs in other studies [17,18,19]. Furthermore, preschool children born in the last quarter of the year are also more likely to be identified as at risk for motor difficulties (amber) and, therefore, need follow-up.

RAE seems to occur, in part, due to maturation differences within the members of the same cohort [15], although it can also affect social or behavioral factors [46,47]. Our results support the idea of Cupeiro et al. [15] since the probability of obtaining a percentile below 5 decreases as preschool children are born earlier in the same year. These relationships and RAE can have consequences, which can be considered negative, in preschool children with less MC, such as less participation in sports activities [21]; a higher rate of abandoning the practice of physical-sports activities [48]; worse physical condition [42]; or less probability of being chosen in sports selection processes [49], which can lead to a sedentary lifestyle [18].

In this sense, we can respond affirmatively to the first secondary objective raised since the MC measured with the MABC-2 battery will be higher in preschool children born in the first quarters of the year compared to those born in the last quarter of the same year, and therefore, those born in the last quarter will be more likely to have a worse classification in the MABC-2 traffic light system.

Regarding gender, both in girls and boys, the percentages of preschool children identified with severe motor difficulties (red; percentile ≤ 5) increase from Q1 to Q4, results that agree with the study by Navarro-Patón et al. [19]. This trend is also present in preschool children identified as at risk of suffering motor difficulties (amber; percentile between 6 and 15), results that agree with those found by Andronikos et al. [50], Birch et al. [51], Brazo-Sayavera et al. [52], and Roberts and Fairclough [53], who point out that the consequences of RAE occur without distinction of gender. This has a special influence on school PE, since if it is not taken into account, it will cause a biased evaluation [52,54], reinforcing the competence of preschoolers born earlier than those born later [21,22] and, consequently, may cause them to obtain lower grades [42,50,51,52].

Analyzing the results of boys and girls within the same quarter of birth, a higher percentage of boys than girls is identified with severe motor difficulties (red; percentile ≤ 5) or at risk of suffering from motor difficulties (amber; percentile between 6 and 15). However, the percentage of boys born in Q1 is higher than that of girls when it comes to identifying preschool children without movement problems (green; ≥16th percentile), being almost three times more likely to do so, compared to girls.

For all the above, according to the second secondary objective, we can indicate that there are differences in MC between boys and girls since the percentage of boys identified with severe motor difficulties (red; percentile ≤ 5) or at risk of suffering from motor difficulties (amber; percentile between 6 and 15) is higher than in girls. However, when it comes to identifying preschool children without movement problems (green; 16th percentile), the percentage of boys born in Q1 is higher than that of girls.

Regarding the limitations of the study, we must indicate that the sample was not selected at random, as well as the geographical distribution, so the results should be taken with caution. On the other hand, we only analyzed the influence of the quarter of birth and no other factors that could influence MC, factors that could explain these differences in preschool children of these ages. Therefore, we want to emphasize that more studies are needed on this subject and we propose as future lines of research to study the set of variables (hours of physical activity in education, hours of extra-curricular physical activity, hours of screen time, family habits of physical activity) that can influence MC in preschool children.

As practical applications, equal opportunities should be provided in educational centers to boys and girls in terms of motor skills. Therefore, RAE should be taken into account when planning, as well as in the evaluation, and during PE sessions.

When planning, the starting point of preschool children must be taken into account and, in this way, structuring school PE in a pedagogically appropriate way, at the initial level of the students. In addition, planning must be done in the short term so that it adapts to the actual development of preschool children and, thus, improves it.

When evaluating, the reference objectives to be achieved must be individualized, evaluating not only the product but also the process. A measure to be applied could be the sensitization of PE teachers about the RAE consequences so that they take them into account when evaluating and applying corrective adjustments [55,56,57].

## 5. Conclusions

Preschool children born in the last trimesters of the year are more likely to have a lower percentile obtained in the MABC-2 battery, and therefore less MC than those born in the first quarters, with RAE existing in the MC of preschool children.

In addition, being a boy and being born in Q1 provides a greater chance of not having MC problems.

## Figures and Tables

**Table 1:** Baseline characteristics.

Variable	Global	Green	Amber	Red
Study population (n, %)	588 (100)	454 (77,2)	74 (12,6)	60 (10.2)
Gender (n, %)				
Male	317 (53.9)	219 (48.2)	50 (67.6)	48 (80.0)
Female	271 (46.1)	235 (51.8)	24 (32.4)	12 (20.0)
Age, median (IQR)	4.66 (4–6)	4.70 (4–6)	4.72 (4–5)	4.33 (4–5.5)
Quarter of birth (n, %)				
Q1.	180 (30.6)	167 (92.8)	9 (5.0)	4 (2.2)
Q2.	121 (20.6)	105 (86.8)	8 (6.6)	8 (6,6)
Q3.	124 (21.1)	88 (71.0)	24 (19.3)	12 (9.7)
Q4.	163 (27.7)	94 (57.7)	33 (20.2)	36 (22.1)
Total percentile Score (0.1–99.9)	51.27 (0.1–99.5)	63.93 (16–99.5)	14.10 (9–15)	1.35 (0.10–5)

Note: Q1: Quarter 1 (Born from January to March); Q2: Quarter 2 (Born from April to June); Q3: Quarter 3 (Born from July to September); Q4: Quarter 4 (Born from October to December). IQR: interquartile range; Green: ≥16th percentile; Children with a normal development. Amber: between 6th and 15th percentiles, both included; Children at risk of suffering motor difficulties. Red: ≤5th percentile; Children with severe motor difficulties.

**Table 2:** Preschool children according to quarter of birth and traffic light zone of the MABC-2 battery.

	Preschool Children (n)	Green (n, %)	Amber (n, %)	Red (n, %)
**Quarter of birth**	(1) Q1. (n = 180)	167 (92.8)	9 (5.0)	4 (2.2)
	(2) Q2. (n = 121)	105 (86.8)	8 (6.6)	8 (6.6)
	(3) Q3. (n = 124)	88 (71.0)	24 (19.3)	12 (9.7)
	(4) Q4. (n = 163)	94 (57.7)	33 (20.2)	36 (22.1)
	**Odds Ratio (CI)**			
	2 vs. 1	0.511 (0.236–1.105)	1.345 (0.504–3.590)	3.115 (0.917–10.586) ***
	3 vs. 1	0.191 (0.097–0.380)	4.533 (2.027–10.139) *	4.688 (1.475–14.896) **
	4 vs. 1	0.107 (0.056–0.203)	4.795 (2.217–10.372) *	12.402 (4.305–35.722) *
	3 vs. 2	0.369 (0.192–0.709)	3.420 (1.471–7.954) **	1.527 (0.601–3.877)
	4 vs. 2	0.206 (0.112–0.379)	3.617 (1.605–8.150) *	4.039 (1.803–9.051) *
	4 vs. 3	0.557 (0.339–0.916) ***	1.058 (0.588–1.902)	2.646 (1.313–5.333) **

Note: Q1: Quarter 1 (Born from January to March); Q2: Quarter 2 (Born from April to June); Q3: Quarter 3 (Born from July to September); Q4: Quarter 4 (Born from October to December); * = *p* < 0.001; ** = *p* < 0.005; *** *p* < 0.05.

**Table 3:** Preschool children according to gender, quarter of birth, and traffic light zone of the MABC-2 battery.

Gender	Children Calification (n)	Green (n, %)	Amber (n, %)	Red (n, %)
**Boys**	(1) Q1. (n = 104)	99 (95.2)	5 (4.8)	0 (0.0)
	(2) Q2. (n = 45)	29 (64.4)	8 (17.8)	8 (17.8)
	(3) Q3. (n = 71)	44 (62.0)	15 (21.1)	12 (16.9)
	(4) Q4. (n = 97)	47 (48.4)	22 (22.7)	28 (28.9)
	**Odds Ratio (CI)**			
	2 vs. 1	0.09 (0.03–0.271) *	4.28 (1.32–13.92) ***	1.22 (1.06–1.39) *
	3 vs. 1	0.08 (0.03–0.23) *	5.30 (1.83–15.36) **	1.20 (1.08–1.37) *
	4 vs. 1	0.05 (0.02–0.13) *	5.81 (2.10–16.05) *	1.41 (1.24–1.60) *
	3 vs. 2	0.90 (0.41–1.95)	1.24 (0.48–3.21)	0.94 (0.35–2.52)
	4 vs. 2	0.52 (0.25–1.07)	1.36 (0.55–2.34)	1.88 (0.78–4.53)
	4 vs. 3	0.58 (0.31–1.07)	1.09 (0.51–2.30)	1.99 (0.93–4.27) ***
**Girls**	(1) Q1. (n = 76)	68 (89.4)	4 (5.3)	4 (5.3)
	(2) Q2. (n = 76)	76 (100)	0 (0.0)	0 (0.0)
	(3) Q3. (n = 53)	44 (83.0)	9 (17.0)	0 (0.0)
	(4) Q4. (n = 66)	47 (71.2)	11 (16.7)	8 (12.1)
	**Odds Ratio (CI)**			
	2 vs. 1	0.89 (0.83–97) **	0.947 (0.89–0.99)	0.947 (0.89–0.99)
	3 vs. 1	0.58 (0.21–1.63)	3.63 (1.05–12.50) ***	0.94 (0.90–0.99)
	4 vs. 1	0.29 (0.12–0.73) ***	3.55 (1.07–11.75) ***	2.45 (0.70–8.54)
	3 vs. 2	1.20 (1.07–1.36) *	1.20 (1.07–1.36) *	-
	4 vs. 2	1.40 (1.20–1.64) *	1.20 (1.08–1.34) **	1.14 (1.04–1.24) **
	4 vs. 3	0.51 (0.21–1.24)	0.98 (0.37–2.57)	1.14 (1.04–1.24) **

Note: Q1: Quarter 1 (Born from January to March); Q2: Quarter 2 (Born from April to June); Q3: Quarter 3 (Born from July to September); Q4: Quarter 4 (Born from October to December); * = *p* < 0.001; ** = *p* < 0.005; *** *p* < 0.05.

**Table 4:** Preschool children according to quarter of birth, gender, and traffic light zone of the MABC-2 battery.

Quarter	Gender (n)	Green (n, %)	Amber (n, %)	Red (n, %)
**Quarter 1**	**(1) Boys** (n = 104)	99 (95.2)	5 (4.8)	0 (0.0)
	**(2) Girls** (n = 76)	68 (89.4)	4 (5.3)	4 (5.3)
	**Odds Ratio**			
	2 vs. 1	2.33 (0.73–7.42) **	0.91 (10.24–3.50)	0.95 (0.89–0.99) **
**Quarter 2**	(1) Boys (n = 45)	29 (64.4)	8 (17.8)	8 (17.8)
	(2) Girls (n = 76)	76 (100.0)	0 (0.0)	0 (0.0)
	**Odds Ratio**			
	2 vs. 1	1.55 (1.25–1.93) *	1.22 (1.06–1.93) *	1.22 (1.06–1.93) *
**Quarter 3**	**(1) Boys** (n = 71)	44 (62.0)	15 (21.1)	12 (16.9)
	**(2) Girls** (n = 53)	44 (83.0)	9 (17.0)	0 (0.0)
	**Odds Ratio**			
	2 vs. 1	0.33 (0.14–0.79)	1.31 (0.52–3.27)	1.20 (1.08–1.33) *
**Quarter 4**	**(1) Boys** (n = 97)	47 (48.5)	22 (22.7)	28 (28.8)
	**(2) Girls** (n = 66)	47 (71.2)	11 (16.7)	8 (12.1)
	**Odds Ratio**			
	2 vs. 1	0.38 (0.19–0.74)	1.47 (0.66–3.27)	2.94 (1.24–6.95) **

Note: * = *p* < 0.001; ** *p* < 0.05.

**Table 5:** Regression models based on the quarter of birth and gender variables.

Traffic Light Color					95% C.I.	
	Model	Chi^2^ Wald	*p*-Value	Exp. (B)	Higher	Lower
Red	Q4	22.297	<0.001	13.028	4.852	37.814
	Q3	7.082	0.008	4.852	1.516	15.529
	Q2	4.998	0.025	4.104	1.190	14.150
Amber	Q4	15.919	<0.001	4.832	2.229	10.475
	Q3	13.809	<0.001	4.624	2.062	10.369
Green	Boy	22.704	<0.001	2.986	1.904	4.682

Note: Q1: Quarter 1 (Born from January to March); Q2: Quarter 2 (Born from April to June); Q3: Quarter 3 (Born from July to September); Q4: Quarter 4 (Born from October to December).

## Data Availability

The data presented in this study are not available in accordance with Regulation (EU) of the European Parliament and of the Council 2016/679 of 27 April 2016 regarding the protection of natural persons with regard to the processing of personal data and the free circulation of these data (RGPD).
